# The Dentist and the Cancer Problem*Read before the Los Angeles County Dental Society, May 16, 1916.

**Published:** 1916-07

**Authors:** Ralph L. Byrnes

**Affiliations:** Professor of Pathology, Medical Department, University of Southern California


### THE DENTIST AND THE CANCER PROBLEM.*

BY RALPH L. BYRNES, M.D.,
Professor of Pathology, Medical Department,
University of Southern California.

At the present time, when the medical societies of the world are most actively interested in carrying on the propaganda for a widespread education of the laity in regard to cancer, naturally the attention of every scientifically inclined person is directed to this disease of the past centuries, to see just what sort of problem confronts him and how great a working basis is given him upon which to start his studies.

In order that you may not consider this the agitation of a group of alarmists or the passing hysteria of a moment, I want to give you by way of introduction a meager resume of the toll of cancer in the last few years, and then take up with you more in detail some of the phases of the disease which confront you as members of the department of oral and dental medicine.

Statistics show that in the United States the mortality from cancer has increased 800 per cent. in sixty-five years,

* Read before the Los Angeles County Dental Society, May 16, 1916.

and in certain parts of England the increase has been 700 per cent in fifty years. (J. H. Kellog, *Public Health Magazine, Michigan.*) In the year 1913 in the registered areas of the United States, 75,000 people died of cancer. As the registered area includes only about sixty per cent. of the population, the number of deaths annually must be much greater than 75,000. Of this 75,000 lives given over to this one disease in one year, the majority are adults, vigorous, healthy breadwinners of this country. The mother is the one to succumb to cancer of the uterus. Usually it is the well-nourished woman who gets cancer of the breast. It is the vigorous man over forty who develops cancer of the lip, tongue or stomach. These facts on which all authorities agree show us the economic value of the warfare on cancer.

From an historical standpoint, cancer and its treatment is a matter of centuries rather than decades; mysticism and quackery played a strong part in its earliest years, nevertheless it was recognized as a serious affection, and one which required most vigorous methods for its extermination.

Strange as it may seem, we are unable to name one individual who alone stands prominent or whose work is monumental in this struggle, and while the past thirty-five years have seen the greatest advances made in the practical studies of the disease, we must remember that this was the age of anesthetics and asepsis, and the results attained by the few men who have accomplished noteworthy results have been indebted more to the material at their disposal, rather than through discovery. "Hippocrates (460 B. C.) says Blair, advised the use of the actual cautery. For twenty centuries this was abused; Butlin opposed it, but now it is again in use, Percy of our own time having demonstrated that heat can be made to deeply penetrate cancerous tissue."

In the sixteenth century the tongue was sutured for injury by Ambroise Peré; but not until the nineteenth were the tissues of the tongue sutured, after the removal of a tumor, when Von Langenbeck performed that operation.

In the seventeenth century an instrument was used to control hemorrhage from the tongue, but until the nineteenth the cautery and ice were the usual hemostatics used.

The first clear description of cancer was given by Wiseman in 1671, but it was not until the middle of the last century that it was clearly differentiated from syphilis. It was the microscope that finally distinguished carcinoma from lymph-angioma and macroglossia. In the eighteenth century several successful operations for the cure of cancer were reported, and at this time Heister of Helinstadt warned that the disease must be clearly excised to include healthy tissue, otherwise it would return worse than before.

Modern operative methods began with C. J. Von Lagenbeck, who in 1819 applied wedge-shaped excisions similar to those used for cancer of the lip, with immediate suture.

It is true that up to the last half of the nineteenth century the treatment of mouth lesions did not keep pace with medical progress along other lines, but this seems to have been due more to lack of co-ordination than lack of individual observations or ideas.

Almost a quarter of a century ago Butlin used, for probably the first time, the term precancerous in referring to certain lesions of the tongue which showed a particular predisposition to change from a benign to a malignant growth. In recent years Bloodgood, in a series of articles on the cancer problem points out that out of 280 pathologically fully developed cancers of the skin and the visible mucous membranes he was unable to find a single case which showed the absence of a previous defect which might be looked upon as a precancerous lesion. W. J. Mayo says: "All vertebrate animals suffer from cancer in situations which are affected by their habits or conditions of life leading to local lesions in the protective mechanism." We must, therefore, look upon local lesions as an invitation to cancer without regard to just what the actual cause of cancer may be.

So far as is known, nowhere else, unless, perhaps, in the stomach, are the majority of clinical carcinomata preceded by recognizable lesions, and in no place can these precancerous or early cancerous conditions be observed as well as in the mouth, where they often persist for years before showing malignancy.

All authorities are agreed that the cures of cancer of the mouth should be the practical as well as the theoretical result. The greatest difficulty is the lateness at which treatment is started.

Considering, on the one hand, its invariable outcome unless successfully operated upon, the sufferings of the victim and the improbability of successful treatment in later stages; on the other hand, the success, or long periods of immunity that may follow comparatively simple early operation; the importance of very early diagnosis can not be overestimated. Therefore, aside from the elimination of predisposing factors, the most important step is the education of physicians, dentists and the public to the necessity of early recognition. It would seem that the weight of this responsibility rests upon the dentist, for there are few persons in this country who do not consult a dentist, the majority of them repeatedly.

Statistics show that the age of the cancer patient is between forty and sixty years, and the predisposition increases with age. One author states that but thirty cases are reported of patients below thirty years of age. Of 207 cases reported by Meller from the Second University Clinic in Vienna between 1894-1904 there were 194 or 94 per cent. males, and 13 or 6 per cent. females.

Practically all authorities agree that carcinoma of the oral cavity is due to irritation of some sort, and we shall go rather extensively into this theory which promises to be our soundest working basis.

Beckman, of the Mayo Clinic, says: ''A cancer on the surface of the body never develops except at a point subjected to continuous irritation for a considerable length of time.''

Bloodgood says: "Cancer never begins in a healthy spot.'' In those areas accessible to sight and touch, we are always informed of a defect entirely different in appearance and size from the cancer which later developed in this spot.''

W. J. Mayo says: ''We do not know the actual cause of cancer, but we do know that it has its soil in a disturbance of the protective mechanism, usually of the skin or mucous membrane of the body. All vertebrates suffer with cancer always in situations in which their habits expose them to chronic irritation. In considering human beings, the evidence as to the relation of chronic irritation to the development of cancer is overwhelming.''

Mayo divides the sites of local irritation into three general groups:

1. Congenital or acquired neoplasms, such as moles, warts and benign tumors of various sorts which may undergo malignancy. Bloodgood refers to papillomatous growths as malignant warts.

2. Trauma: Coley, in an analysis of 250 cases of carcinoma which came under his personal observation, and the histories of which were taken by himself, found a history of antecedent trauma in 32.8 per cent. of the cases. This theory is also supported by McWilliams, Von Bergmann, Ropke, Murphy, Ziegler, Lowenthal, Liebe and Von Graefe.

3. Chronic irritation: Whether the result of mechanical, chemical or infectious agencies, chronic irritation is the most important of all those precancerous conditions with which we are acquainted, and it is undoubtedly the most potent influence in the development of the disease following congenital lesions and trauma. This third division is the one which interests us principally in our consideration of cancer of the oral cavity. Under infectious agents we may perhaps place syphilis as the most important of this group. Under chemical agencies may be mentioned the development of cancer following the chemical irritation caused by tar, paraffine, petroleum, arsenic and aniline products. The development in local lesions produced by heat as from smoking.

Under mechanical agencies Taylor mentions tobacco smoking as first in importance of the etiological factors of carcinoma of the oral cavity—aside from the chemical irritation of the tobacco smoke and juice, the physical irritation of the pipe, due either to the roughness of the pipe stem or because of its being a foreign body held in the same place, produces a constant irritation.

The second and to us the most important mechanical agent is the irritation caused from broken or decayed teeth, acting on the tongue or cheek, or the irritation caused from broken or badly fitting dental plates, crowns or bridges. One of the three most important etiological factors in cancer of the tongue as given by Taylor is the irritation from a broken or jagged tooth. Ehrlich found broken or carious teeth in only ten per cent. of the cases in his series—most observers, however, believe it an important factor. Kummel considers broken and decayed teeth to be more often a factor in cancer of the cheek than of the tongue. He says the growth is especially apt to start well back on the cheek opposite a molar or wisdom tooth and to extend to the anterior pillar of the fauces and from there to both the upper and lower jaws, limiting their movements. It rarely perforates the cheek. It starts as an ulcerated area and frequently opposite a broken tooth.

Blair says: "Of the tumors which arise within the mouth most of them seem to be in connection with dental or mechanical irritation. In tumors arising in the antrum or nasal cavity pain is an early and almost constant symptom; this may be localized in the form of toothache or diffused over the distribution of the fifth cranial nerve."

All cancers in their early stages are localized to small areas and can nearly always be cured. Beckman says that we must educate the public to realize that any ulcer of the lip, and we may include cheek and tongue, which does not heal readily under treatment, is almost surely a cancer, and should be removed at once for microscopical examination, and if this examination reveals the presence of carcinomatous change, then a radical operation should be done.

Butlin, one of England's foremost men in the study and treatment of cancer, after devoting twenty-five years to his work, stated in 1909 that he had operated upon only a little more than 200 cases of cancer of the tongue during that quarter of a century due to the lateness of the presentation of the cases.

In the United States previous to 1910 the Massachusetts General Hospital reported that of 172 cases of cancer of the mouth treated there twenty-nine per cent. were inoperable. Later statistics from Johns Hopkins lower this rate.

If we realize that from the registration area of the United States in 1912, 1,838 deaths from cancer of the oral cavity were reported, and remembering that all authorities agree that early diagnosis with early treatment is our one means of preventing this enormous waste of life, we can see that this is indeed a problem and one which confronts every intelligent person, especially the one whose eye and mind are trained along lines which make him a potent factor in this campaign.

You can readily see that it is during the cancer age that the teeth are disintegrating and artificial ones are replacing them, and at this period, if never before, the ordinary individual approaches the dentist.

Kirby-Smith says: "You may have under your care ossifying periostitis of the alveolar border of the jaw, or a beginning epulis, or, rarer still, adamantine epithelioma. These at first often closely resemble an ordinary gum boil, a simple infection around the gum with the formation of an abscess which slowly undergoes resolution. With this condition care in regard to repeated lancing is necessary, for there is always the possibility present that you are dealing with the former conditions instead of with a simple infection. There is also the possibility that this insignificant swelling may be the beginning of a periosteal sarcoma or a more rapidly growing true carcinoma. Certainly repeated lancing of a beginning malignant growth is to be deplored. A little delay for careful investigation will not do much harm in the case of a simple swelling around the gums which has persisted for some time in spite of ordinary measures, and this delay may be the means of giving your patient a hope of being cured by a complete operation before the cancer has been stimulated to renewed activity through being irritated by incisions.

"The X-ray as a diagnostic measure is of great value to the dentist in those cases of unerupted teeth, alveolar sinuses, necrotic processes of the jaw or abscess of the antrums, and should be resorted to in all doubtful cases."

Until the much desired specific is discovered, it is chiefly from the wideawake dentist, well grounded in oral pathology, who makes a complete examination of the entire mouth of his patient, that the medical profession as well as the public must receive the greatest assistance.

### Bibliography.

Bloodgood, L. C.—"What Everyone Should Know About Cancer," *Michigan Public Health Journal.*

Coley.—*Annals of Surgery,* March, 1898.

Bloodgood, L. C.—*Journal A. M. A.,* Dec. 27, 1913.

Hertzler.—"Treatise on Tumors."

Butlin.—"Diseases of the Tongue."

Da Costa.—"Keen's System of Surgery."

Bland-Sutton.—"Tumors Innocent and Malignant."

Taylor.—"Cancer—Its Study and Prevention."

Blair.—"Surgery—Diseases of the Mouth and Jaws."

Blair.—"Tumors of the Mouth."

Ryall.—*British Med. Journal,* April, 1913.

STEINER.—*Deutsche Zeitschrift fur Chirurgie,* Vol. XCVIII.

EHRLICH. — *Archiv fur Klinisher Chirurgie,* Vol. LXXXVIII.

KUMMEL.—*Krankheiten des Mundes.*

HALLSTROM.—"Operations des Lippen Kreben."

MELLER.—*Deutsche Zeitschrift Chirurgie,* Vol. LXXXIV.

W. J. MAYO.—"The Prophylaxis of Cancer," *Annals of Surgery,* June 14.

W. J. MAYO.—"The Cancer Problem," *Journal-Lancet,* July, 1915.

KIRBY-SMITH.—*Journal Florida Medical Association,* July, 1915.—*Pacific Dental Gazette.*

